# Role of serology tests in COVID-19 non-hospitalized patients: A cross-sectional study

**DOI:** 10.1371/journal.pone.0266923

**Published:** 2022-04-14

**Authors:** Mohammad Taghi Haghi Ashtiani, Parisa Sadeghi Rad, Kosar Asnaashari, Alireza Shahhosseini, Fatemeh Berenji, Setareh Mamishi

**Affiliations:** 1 Children’s Medical Center, Tehran University of Medical Sciences, Tehran, Iran; 2 Pediatric group, faculty of medicine, Tehran University of Medical Sciences, Tehran, Iran; 3 Network of Immunity in Infection, Malignancy and Autoimmunity (NIIMA), Universal Scientific Education and Research Network (USERN), Tehran, Iran; Central laboratory, The Affiliated Yantai Yuhuangding Hospital of Qingdao University, CHINA

## Abstract

**Introduction:**

Severe acute respiratory syndrome coronavirus (SARS-CoV2) has imposed catastrophic impressions on the world. After all the focused researches conducted in the COVID-19 area, many features remain obscure. We have surveyed 1,363 outpatients with suspected COVID-19 in Tehran, Iran. The analysis emphasized on characteristics of patients with positive PCR or serology of SARS-CoV-2.

**Methods:**

The nasopharyngeal swabs were tested for SARS-CoV2 PCR. Serum specimens were tested for SARS-CoV2 IgG and IgM. Clinical presentations of the patients, history of chronic diseases or drug use, contact with a possible COVID-19 patient and previous infection with SARS-COV2 were investigated.

**Results:**

Of the total 1,363 investigated patients, 22% had positive SARS-CoV-2 PCRs, 82% had positive IgG, 38% had positive IgM, and 31% had both positive IgM and IgG values. Positive serologic tests were significantly associated with a positive PCR test obtained previously in the course of the current disease (P value<0.001).

IgG and IgM antibody values were significantly associated with underlying disease, cough, fever, chills, fatigue, and myalgia (all P values <0.001). Dyspnea was significantly associated with IgG levels (P value = 0.01), yet it was not associated with IgM serology (P value = 0.2). Positive serology tests were not associated with symptoms of coryza.

GI symptoms were not associated with positive IgG test (P value = 0.1), yet it did show an association with positive IgM test (P value = 0.02).

Cough, fever, chills, myalgia fatigue, dyspnea, and GI symptoms were all significantly associated with positive PCR (all P values <0.001), and symptoms of coryza did not show a significant relationship (P value = 0.8).

**Conclusion:**

Assessing antibody titers in outpatients is invaluable due to the epidemiological importance of investigations in mild or even asymptomatic cases. Since the number of such studies in non-hospitalized patients is not high, the current study can be used as a comparison model.

## Introduction

Since the emergence of the novel Coronavirus infection, severe acute respiratory syndrome coronavirus (SARS-CoV2), on December 19 in Wuhan, China [1], the world has been shaking by the impacts of the catastrophe. To date (March 2022), more than 455 million confirmed cases of SARS-CoV2 have been detected globally, and it has caused more than 6 million deaths [2].

Most Coronavirus Disease 2019 (COVID-19) patients are reported to manifest self-limited symptoms. Fever, cough, fatigue, myalgia, gastrointestinal (GI) symptoms, headache, and loss of taste and smell are the most common reported symptoms [3–5]. However, the rapid spread of the disease has made the total number of patients presenting severe or life-threatening conditions considerably high. Cardiac complications like arrhythmia and acute cardiac injury, acute respiratory distress syndrome, thrombotic complications, acute kidney injury, sepsis, and shock, are introduced toas the most serious complications of COVID-19 [6–8]. Novel manifestations are reported as the new cases of infected patients are being diagnosed, and it can be implied that COVID-19 is a disease with various clinical presentations [9–11].

Laboratory evaluations should be performed for clinically suspected cases of COVID-19, including SARS-CoV2 nucleic acid, antibody, or antigen detection tests [12]. Nucleic acid testing, such as real-time reverse-transcription polymerase chain reaction (rRT-PCR), is the first recommended analysis [13]. The virus might be detectable in the upper respiratory tract from three (in mild cases) to fourteen (in severe cases) days before the onset of symptoms [14]. Seroconversion, defined as the presence of measurable antibody response after infection, can be invaluable in the diagnosis of COVID-19 patients. It has been declared that IgG and IgM antibody levels increase faster in settings of more severe disease, in comparison with asymptomatic infections or milder presentations. Seroconversion can be detected at the end of the first week of the onset of symptoms of COVID-19; however, it might take weeks to develop in patients with milder infection [15–17].

Although about one year and a half have passed since the emergence of the pandemic, many features of COVID-19 are still obscure. Since the introduction of the pandemic, lots of effort and resource have been donated to find a cure for this catastrophic disease. Various treatments proposed have not been able to restrain the rebellious nature of the disease [18–20]. Even though three doses of vaccines have been administered in most regions of the globe up to now, thousands of people are dying each day due to the complications of COVID-19 [2]. The huge burden of this disease makes investigations about it vital.

We have surveyed 1,363 patients suspected of being infected with Covid-19, who were referred to two laboratories for COVID-19 serology and/or RT-PCR test in Tehran, Iran. The analysis emphasized on characteristics of patients with positive PCR or serology of SARS-CoV-2, and we aimed to find out if certain symptoms or characteristics of clinically suspected COVID-19 patients were associated with PCR and serology status of the patients. We hope these surveys can enlighten the path to better understanding and management of the pandemic.

## Methods

Our study investigated a total of 1,363 non-hospitalized clinically suspected COVID-19 patients who were referred to Children’s Medical Center laboratory (137 patients) and a private laboratory in Tehran, Iran. These patients were referred to the laboratories for PCR and/or serology tests of COVID-19 by their physicians. They were suspected to be infected with COVID-19 due to the presence of COVID-19 symptoms, i.e., cough, fever, fatigue, chills, myalgia, loss of smell and appetite, GI symptoms, and dyspnea. The patients with primary or secondary immunodeficiency disorders were excluded. It should be noted that none of the patients had received COVID-19 vaccination before participating in the study. Trained laboratory staff collected the nasopharyngeal swabs, which were tested for SARS-CoV2 rRT PCR (Pishtaz-Teb one-step RT-PCR kit). Serum specimens were collected and Pishtaz-Teb Elisa kits were used to test for SARS-CoV2 IgG and IgM. Clinical presentations of the patients, history of chronic diseases or drug use, contact with a possible COVID-19 patient and admission due to previous infection with SARS-COV2 were investigated in patients with positive or negative serology tests. Data analysis was performed via SPSS.

### Ethics

Informed written consent was obtained from the participants of the study, and from the parents when the patient’s age was less than 18. The study adhered to the tenets of Helsinki declaration [21] and the ethical committee of Children’s Medical Center, Tehran University of Medical Sciences, Tehran, Iran, and was approved by the ethical committee of Children’s Medical Center, Tehran, Iran. The ethical code is IR.TUMS.CHMC.REC.1401.010.

## Results

**Fig 1:**
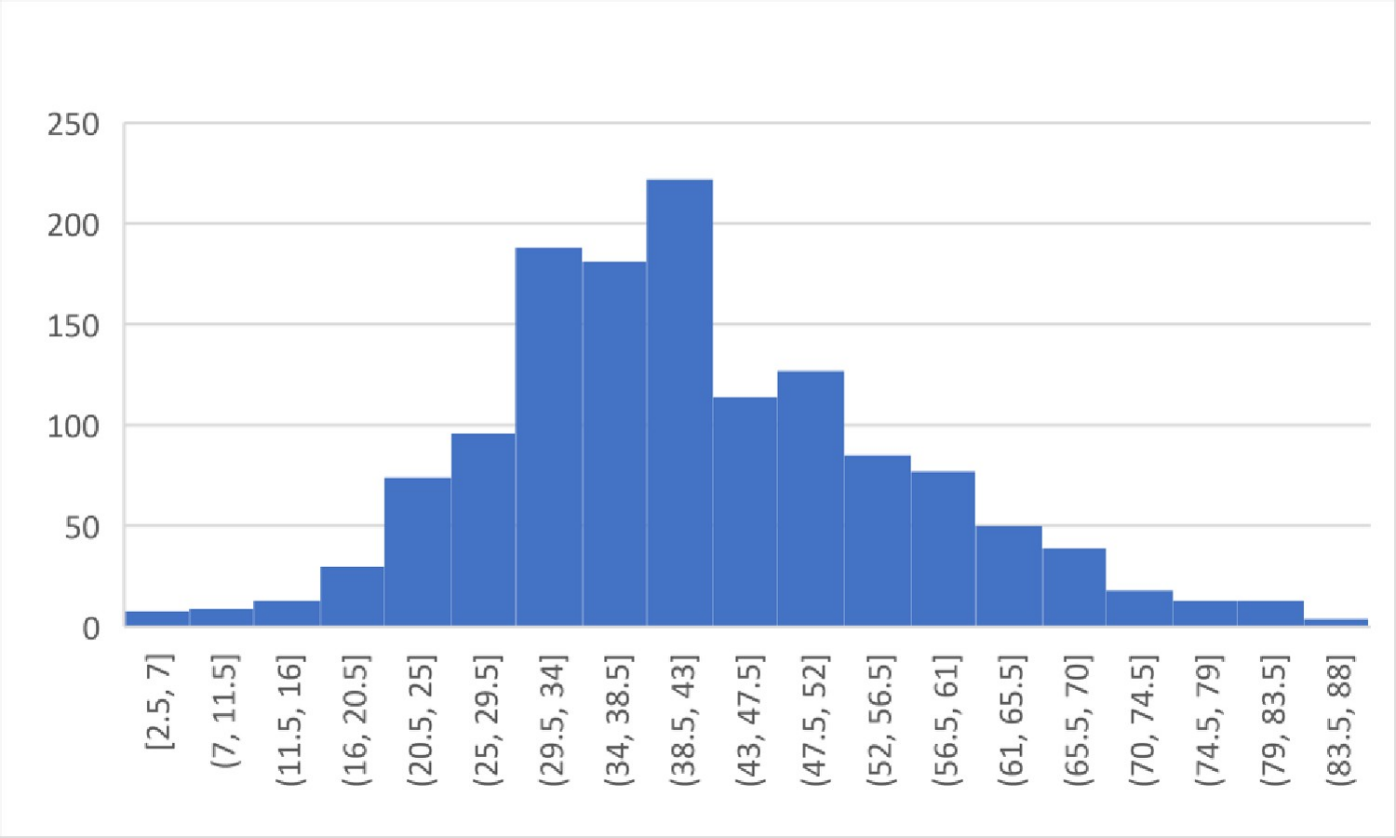
Age distribution of the participants.

We surveyed 1,363 patients clinically suspected to be infected with SARS-CoV-2. These patients were referred to the Children’s Medical Center laboratory (137 patients) and a private laboratory in Tehran, Iran, for COVID-19 PCR and serology tests. Fifty-one percent of the patients were males and forty-nine percent were females. The age of the patients ranged from 2 to 88 years, and the mean age was 42 ± 14.2. The age distribution is depicted in Fig 1.

The laboratory evaluations were conducted with a mean interval of 23 ± 7.3 days from the onset of the symptoms. Underlying conditions such as diabetes, cardiovascular disease, hypertension, or other chronic diseases such as renal, thyroid, respiratory, or neurologic diseases were assessed. Patients’ characteristics are summarized in Table 1.

**Table 1:** Characteristics of the patients.

Characteristic	Frequency
History of chronic diseases	4%
History of contact with COVID-19	45.2%
Previous admission due to COVID-19	5.1%
Cough	34.6%
Fever	37.5%
Chill	30.7%
Fatigue	41.2%
Myalgia	42.7%
Dyspnea	18.5%
Coryza	34.5%
Gastrointestinal symptoms	17.3%

Of the total investigated patients, 82% had positive IgG and 38% had positive IgM levels. Out of the total number of patients, 435 (31%) had both positive IgM and IgG values.

Male patients tended to be more IgG-positive than females (P value<0.001); such a gender-based difference was not observed in IgM positive group.

**Fig 2:**
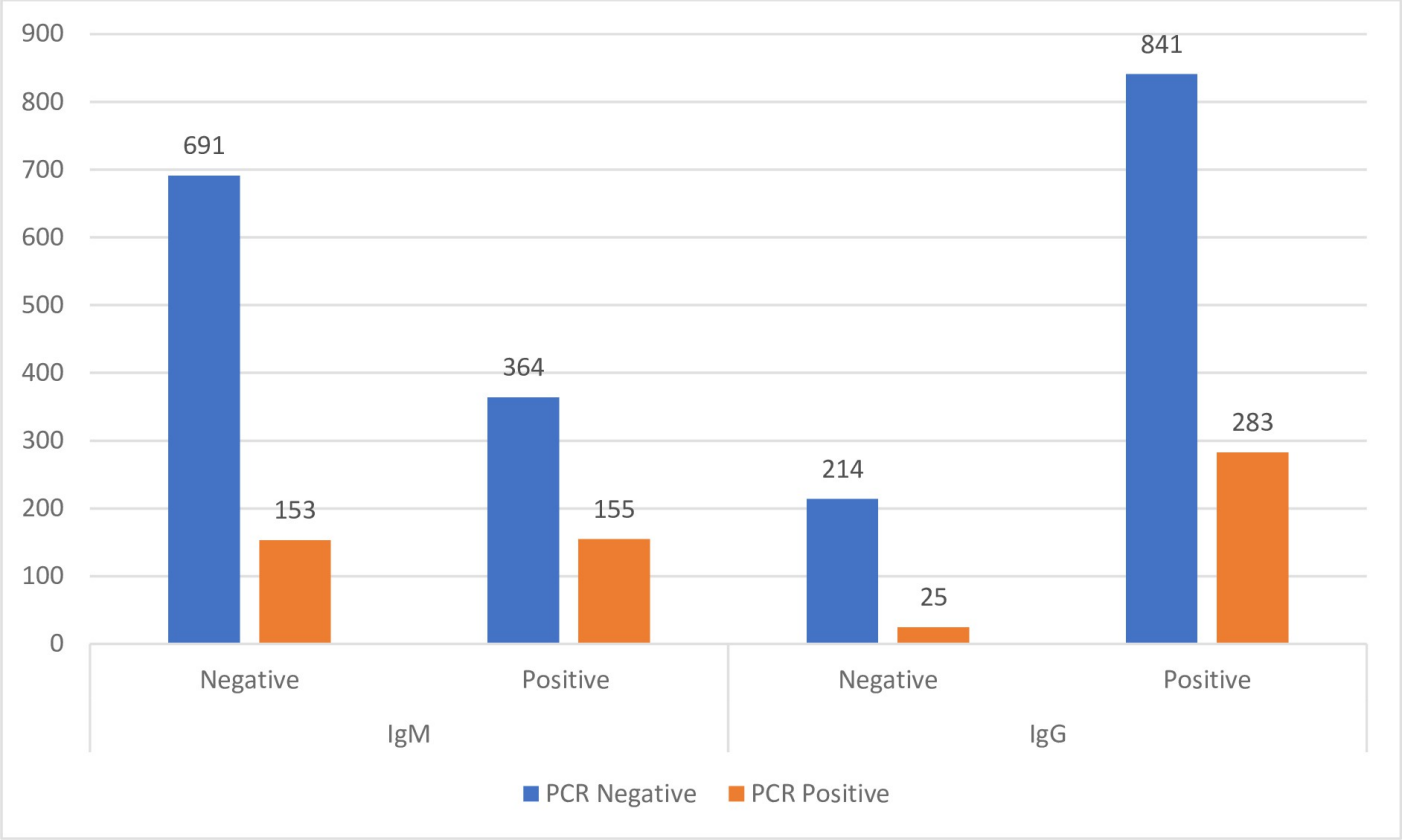
The distribution of SARS-CoV-2 serology groups in PCR positive and negative patients.

Positive IgM and IgG tests were significantly associated with a positive PCR test (P value<0.001). It should be noted that specimens for PCR and serology tests were not obtained necessarily at the same time, since the patients were referred for serologic tests and PCR testing might have had already been performed. Fig 2 shows the distribution of SARS-CoV-2 serology groups in PCR positive and negative patients.

The values of positive serology tests, both IgG and IgM, were significantly higher in patients with underlying disease (P value<0.001), cough (P value<0.001), fever (P value<0.001), chills (P value<0.001), fatigue (P value<0.001), and myalgia (P value<0.001). Dyspnea was significantly associated with IgG levels (P value = 0.01), yet it was not associated with IgM serology (P value = 0.2). Positive serology tests were not associated with symptoms of coryza.

While positive IgG test was not shown to be associated with GI symptoms (P value = 0.1), there was an association between positive IgM test and GI symptoms (P value = 0.02).

The patients with a positive contact history with COVID-19 at home or work were more probable to have a positive IgG (P value<0.001); however, home contact history was not shown to be associated with positive IgM results (P value = 0.7).

Twenty-two percent of investigated patients had positive SARS-CoV-2 PCR. Cough, fever, chills, myalgia fatigue, dyspnea, and GI symptoms were all significantly associated with positive PCR (all P values<0.001), and symptoms of coryza did not show a significant relationship (P value = 0.8). There was a significant association between positive PCR and history of contact with a COVID-19 positive person at home or at work (P value<0.001). The distribution of symptoms within groups with positive and negative PCR and serology is shown in Table 2.

**Table 2:** Distribution of symptoms within groups with positive and negative PCR and serology.

		Cough	Fever	Chills	Fatigue	Myalgia	Dyspnea	Coryza	GI symptoms
**PCR Positive N = 308**	Symptom within the group	46%	52%	43%	55%	57%	26%	35%	25%
**PCR negative N = 1055**	Symptom within the group	31%	33%	27%	37%	38%	15%	33%	14%
**P value**		<0.001	<0.001	<0.001	<0.001	<0.001	<0.001	0.8	<0.001
**IgM Positive N = 519**	Symptom within the group	40%	45%	36%	47%	48%	31%	31%	20%
**IgM negative N = 844**	Symptom within the group	31%	33%	27%	37%	39%	36%	36%	15%
**P value**		<0.001	<0.001	<0.001	<0.001	<0.001	0.18	0.1	0.02
**IgG Positive N = 1124**	Symptom within the group	37%	41%	33%	44%	46%	19%	35%	18%
**IgG negative N = 239**	Symptom within the group	23%	20%	16%	27%	25%	12%	31%	12%
**P value**		<0.001	<0.001	<0.001	<0.001	<0.001	0.01	0.2	0.1

There was no significant relationship between age and positive PCR, yet age showed an association with SARS-CoV-2 IgG levels (P value = 0.02).

## Discussion

Despite devoting more than 2 years of focused global research to COVID-19, there are still lots of mysteries. The role of antibodies in disease modification is one of the puzzling concepts. The current study investigates a relatively large population of outpatients with symptoms of COVID-19, emphasizing on their serology status and the correlations between the serology and PCR test, as well as the patients’ symptoms and characteristics.

In a study conducted by Lombardi et al in Italy, November 2020, the seroprevalences of anti-SARS-CoV-2 IgG of 4055 healthcare workers were assessed and a sensitivity of 97.4% and a specificity of 98.5% were reported [22]. This study highlights the importance of checking IgG levels in diagnosing infection with COVID-19. Similar to our study, Lombardi’s study showed an association between age and positive serology (P value = 0.02) in which patients younger than 30 and older than 60 years were more probable to have positive IgG values.

Our study showed that IgG levels, unlike IgM, were higher in male patients; However, the female or male preponderance has revealed different results in different studies, some not being able to find a difference and some finding that one gender is associated with positive serology [22,23].

Wolfel’s study in May 2020 showed that PCR testing on simple throat swabs in the early stages of COVID-19 provides sufficient sensitivity for detection of the disease, compared with testing on sputum, serum, urine, or stool. After 14 days of disease onset, seroconversion could be found for IgM and IgG in all patients [14]. In a study conducted by Deng et al in August 2020, it was demonstrated that non-human primates develop effective antibody responses against SARS-CoV-2, which makes them resistant to reinfection [24]. Hassan et al published a study in August 2020 showing that passive transfer of sera containing a neutralizing monoclonal antibody leads to a reduced viral load of SARS-CoV-2 in mice [25]. The role of SARS-CoV-2 antibodies in active coronavirus infections in humans is still under debate.

In June 2020, Long et al investigated 285 hospitalized patients with positive SARS-CoV-2 PCR and declared that all of the patients were found to be IgG positive 17–19 days after symptom onset. On the other hand, IgM antibody level reached a peak of 94.1% on days 20–22 [26]. In the present study, 50% of the patients with positive PCR had positive IgM tests, and 92% of the positive PCR patients were IgG positive. There was a positive association between COVID-19 serology and PCR tests (P value<0.001). Since some of the patients were referred to check the serology status and their PCR tests had been performed before the referral, the exact interval between symptoms’ onset and PCR test was missing in some patients.

PCR tests can detect a current infection with SARS-CoV-2, while serology tests report a past exposure. Serology tests can inform us about those patients with neutralizing antibodies who are thought to be protected against reinfection and can potentially return to work [27]. In the report published by WHO in September 2020, it was discouraged to use serology testing as a way to diagnose COVID-19, and suggested to use it only to determine previous infection. Nevertheless, this application is not quite beneficial except for epidemiological surveys, since the time for intervention would have already passed [13]. Studies are still trying to evaluate the role of antibody detection in diagnosis of COVID-19.

SARS-CoV2 IgG levels can be found in patients as soon as one week from the disease onset and have shown positive yields in up to 100% of hospitalized patients until the third week of symptom presentation, but the duration for which antibody levels are elevated is not definite [15,28]. The Cochrane meta-analysis conducted in April 2020 surveyed all the published data on COVID-19, i.e., 8526 cases of SARS-Cov-2 globally. It concluded that sensitivity of antibody tests is low in the first week, and seroconversion is not suitable as an early method for diagnosis of COVID-19. Nevertheless, serology can be invaluable in diagnosis later in the course of the disease, or when RT-PCR test is not done or is negative. The sensitivity was found to be higher after 15 days of disease onset, yet there was not a consensus on the duration of antibody rise. Since the surveyed studies were mainly conducted in hospital settings, it was unclear whether serology tests are efficient in detecting lower antibody levels assumed to be detected in mild COVID-19 disease [15]. In the present study, 82% of patients had positive IgG, 38% had positive IgM and 31% had positive serology for both, pointing out the long interval between testing and the presence of symptoms.

In a study conducted by Ravensburg et al in June 2020 on 111 outpatients with suspected COVID-19 disease, serum samples were collected between days 10 to 68, and 81.1% of the patients had positive SARS-CoV-2 IgG levels. Interestingly enough, seropositivity was not different in patients with less or more than 20 days of symptoms [29].

Critically ill patients are reported to have higher titers of SARS-CoV2 IgG in comparison with patients with less severe disease. That is why we can hypothesize that non-hospitalized patients, i.e., the patients with less severe symptoms, might have lower titers of SARS-COV-2 IgG. In the present study, conducted on outpatients referred to laboratories for PCR and serologic evaluations of COVID-19, 50% of the patients with a history of positive PCR in the course of the disease showed positive IgM tests, and 92% of the positive PCR patients were IgG positive. There was an affirmative association between COVID-19 serology and PCR tests (P value<0.001).

## Conclusion

Assessing antibody titers in COVID-19 outpatients is invaluable due to the epidemiological importance of investigations in cases with mild disease or even asymptomatic ones. As the number of patients with mild COVID-19 disease is considerably high, a better understanding on when to check antibody titers can save substantial resources. Since there are not many studies conducted on non-hospitalized patients, the current study can be used as a comparison model.
